# On the Action of the Bromide of Potassium in Inducing Sleep

**Published:** 1864-09

**Authors:** Henry Behrend


On the Action of the Bromide of Potassium in Inducing Sleep.—*By Henry Behrend, I. R. C. P. E. etc.*— Dr. Garrod, in his recent lectures on the British Pharmacopœia, has mentioned that the bromide of potassium, when administered in large doses, produce drowsiness. I do not know whether the profession at large is aware of this fact, but as I have never previously seen any record of it (being indebted for my first information on the subject to the statements of Dr. Brown-Sequard, and as I have, during the past twelvemonth, had ample practical experience of its use, the following cases are submitted to demonstrate the value of the remedy in the treatment of insomnia and restlessness, accompanied by and dependent upon nervous excitement and irritability. If its employment upon a larger scale should confirm the results at which I have arrived (and of which Dr. Brown-Sequard has repeatedly assured me,) its importance can not well be overrated; as it is better borne than opium or any of its preparations, is free from the unpleasant effects—such as headache, constipation, &c.,—produced by that drug, and the system does not so rapidly become accustomed to it as to require its administration in constantly-increasing doses.

The first case in which I prescribed it was that of a gentleman, thirty-six years of age, of highly nervous temperament, who had undergone much mental excitement consequent upon the dangerous illness of a very near relative. There was no constitutional malady present, and the only symptom was loss of sleep, and the debility, both bodily and mental, consequent upon it. He had not enjoyed really a good night for weeks, and this preyed upon him to such an extent, as almost to preclude the possibility of his sleeping; for his mind was constantly intent upon this one subject, and never more so than when he retired to rest, so that it seemed as if the very effort to obtain sleep prevented its accomplishment. He was in very low spirits, and had failed in quieting the nervous system by opium in its various forms, valerian, and other antispasmodics and sedatives. He was recommended to take twenty-five grains of the bromide of potassium dissolved in a little cold water three times a day, before meals, for a week. At the end of this time, he called to inquire if it was necessary to continue the treatment, as he had enjoyed several night's excellent sleep, and had to a considerable extent regained his former cheerfulness and mental calibre. As he was still, however, somewhat nervous about his night's rest, it was thought advisable that he should not entirely give up the employment of the bromide; and he continued taking it once in the twenty-four hours, at bedtime, for a fortnight longer. He had now implicit confidence in the power of the remedy, and, what was of still greater consequence, was regaining confidence in his own powers of obtaining natural sleep, and he gradually ceased having resource to the medicine. He always, however, kept a dose of it by his bedside, so that if he woke in the night and was tormented by the fear of not sleeping again, he might at once take it. During the last few months this fear has left him, and he does not now use the bromide on the average more than once in three weeks. He sleeps perfectly well for six or seven hours at a time, and wakes comfortably and naturally, with entire freedom from the dread and depression which he formerly experienced on waking.

A second case, perhaps even more remarkably illustrative of the beneficial action of this salt, is that of a gentleman, forty years of age, who consulted me in the month of October last. He was of a most excitable and nervous temperament, and was engaged in mercantile transactions of great magnitude, the extent of which indeed seemed quite to overwhelm him, although without any grounds as to a fear of their ultimate result in a pecuniary point of view. He was quite unable however, to banish them from his mind day or night; he had lost his natural sleep, was harassed and fatigued during the day, and sought my opinion as to whether he ought not at once to withdraw from business, although the sacrifice entailed thereby would be very great, and he was most anxious to avoid it. I told him to place himself under treatment for a few weeks, and if no benefit were derived at the end of that time, such a step as he contemplated might be necessary. I prescribed the bromide of potassium as in the last case: twenty-five grains to be taken three times a day before meals. At the end of a week he was much better, slept naturally and well, and was consequently much more sanguine as to his capability of attending to his affairs. Good sleep having been procured, I thought it better to attend to the condition of the nervous system, and ordered the sulphate of strychnia to be taken in commencing doses of the thirtieth of a grain, to be gradually increased to the tenth of a grain, thrice daily. He was advised to have a dose of the bromide of potassium by his bedside, or to take one before going to bed, if he felt nervous about his night's rest; but since the first week of the treatment I do not think he has once found it necessary to have recourse to it. He sleeps perfectly well, has regained spirits and confidence, and has quite abandoned the idea of his unfitness to attend to his business transactions. He continues taking the tenth of a grain of sulphate of strychnia twice daily.

Other instances might be adduced of a similar character, but the above will serve as a type of the cases in which the administration of the bromide of potassium appears likely to be most useful—those, namely, in which the nervous element preponderates; and it is in these that, for the most part, opium and its preparations fail to produce any good result, and are not well borne by the system, frequently even adding to the excitement and irritability under which the patient labors. There can be no doubt, moreover, that cases of this type are unfortunately on the increase, since the highly artificial mode of life of the present day, especially in large cities, perpetually stimulates the nervous energy to the highest possible degree; so that in the strongest constitutions the mental equilibrium is but too often shaken, and the weaker ones yield speedily to the excessive demands made upon them. The dose of the bromide recommended may appear large, but it is in all cases easily tolerated, and produces neither disagreeable nor toxical effects; the appetite is not interfered with, the alvine evacuations are regular and copious, and irritability of the bladder—a frequent accompaniment of restless nights—is greatly relieved. The only unpleasant result I have witnessed has been slight and temporary headache; and Dr. Brown-Sequard has informed me that he has given it with perfect safety for several successive weeks in drachm doses. Of the temporary paralysis, and weakening of sexual desire and power, which are said to follow upon the administration of large doses of the bromide of potassium, I have seen nothing. I should wish to try this remedy in the treatment of the restlessness of delirium tremens, but have not had the opportunity since I have become acquainted with its action upon the nervous system.—*London Lancet.*

